# Sources and Level of Patient Knowledge Regarding Available Prenatal Diagnostic Methods and the Frequency of Their Use in the Polish Population

**DOI:** 10.3390/healthcare13233168

**Published:** 2025-12-04

**Authors:** Małgorzata Świątkowska-Freund, Magdalena Tworkiewicz, Adam Kosiński, Szymon Bednarek

**Affiliations:** 1 The Academy of Applied Medical and Social Sciences, 82-300 Elblag, Poland; a.kosinski@amisns.edu.pl; 2 The Ludwik Rydygier Provincial Polyclinical Hospital in Torun, 87-100 Torun, Poland; m.tworkiewicz@outlook.com (M.T.); prenatalium@gmail.com (S.B.)

**Keywords:** prenatal testing, prenatal screening, prenatal screening coverage, prenatal in Europe, frequency of prenatal, non-invasive testing, invasive prenatal

## Abstract

**Introduction:** The scope and accessibility of prenatal testing have significantly expanded in recent years, reaching a broader population of pregnant women. Advances in non-invasive diagnostic methods support informed decision-making and help reduce the need for invasive procedures. **Objective:** The objective was to evaluate pregnant women’s knowledge regarding prenatal testing and assess the quality of information provided by healthcare professionals, including the frequency of screening and invasive procedures. **Materials and Methods:** A total of 310 obstetric patients from maternity wards in two hospitals in northern Poland completed a survey addressing prenatal tests, sources of information, and the quality of guidance received from medical staff. **Results:** Nearly 75% of respondents demonstrated adequate knowledge of the purpose, indications, and scope of prenatal testing. Physicians were identified as the primary source of information. Approximately 50% correctly indicated the recommended number of ultrasound examinations during pregnancy. No correlation was observed between knowledge of prenatal testing and a history of delivering a child with health complications. The combined first-trimester test was performed in 48.6% of cases, NIPT in 11.6%, and invasive testing in 1.8% of the study group. **Conclusions:** Public awareness of prenatal testing in Poland remains insufficient. With the introduction of partially reimbursed tests in 2024, we recommend strengthening educational efforts through social campaigns and targeted training for healthcare professionals.

## 1. Introduction

Prenatal testing has become increasingly accessible to a broader population of pregnant women as costs decrease and availability improves. While non-invasive tests offer significant advantages, they are not entirely risk-free; for example, a false-positive result may lead to an invasive procedure, which carries its own risks. Nevertheless, the primary goal of these methods is to reduce the overall need for invasive testing, thereby minimizing associated complications. The development of non-invasive prenatal diagnostic techniques aims to decrease the number of pregnant women undergoing invasive procedures and to optimize the criteria for such interventions [1,2,3,4,5].

The purpose of prenatal testing extends beyond identifying fetal disorders; it also enables intrauterine treatment for certain conditions. In some cases, this approach can be life-saving—for instance, in twin pregnancies complicated by twin-to-twin transfusion syndrome or in cases of obstructive urinary tract anomalies, diaphragmatic hernia, or large placental vascular malformations. Additionally, prenatal interventions can reduce the severity of organ damage, as seen in spina bifida [6,7,8].

In most countries, including Poland, ultrasound examinations are an integral part of routine screening offered to all pregnant women. International guidelines recommend a first-trimester ultrasound, which not only detects structural anomalies but also estimates the risk of common chromosomal abnormalities. Furthermore, combined testing is performed in accordance with the Fetal Medicine Foundation guidelines [9,10]. In selected cases, non-invasive prenatal testing (NIPT) may also be offered during this period, although recommendations vary considerably across countries [11,12,13,14,15,16,17].

These tests are particularly useful for women with intermediate risk based on combined screening results, but they remain a screening tool rather than a diagnostic one. For patients classified as high-risk through non-invasive testing, invasive diagnostic procedures are recommended. These procedures are diagnostic, not screening, and are reserved for a specific subset of pregnant women [11,18].

The second ultrasound examination is typically performed between 18 and 22 weeks of gestation. However, recommendations regarding third-trimester scans differ significantly among countries, reflecting a lack of consensus [10,19].

In Poland, the Prenatal Testing Program has been in place since 2008, allowing examinations within a defined scope at specialized clinics based on physician referrals [20]. The National Health Fund covers first- and second-trimester ultrasounds and combined tests for patients meeting high-risk criteria for congenital (genetic and structural) anomalies. At the time of this study, women who did not meet program criteria were advised about the option of undergoing combined testing at their own expense. Beginning in June 2024, all pregnant women will be eligible for the program, regardless of their assessed risk of chromosomal abnormalities [21]. However, adapting to new guidelines takes time; physicians must familiarize themselves with updated recommendations and develop the habit of informing patients about available options [22].

Despite clearly defined indications for various prenatal diagnostic methods, many pregnant women in Poland still do not undergo tests recommended by current standards. Regional disparities in program implementation are also evident. For example, according to National Health Fund data from 2022, 5274 patients in the Kuyavian-Pomeranian Voivodeship underwent testing (35% of live births), compared to 2959 women in the Pomeranian Voivodeship out of 20,993 live births (14%) [23].

This study aimed to assess the level of knowledge among pregnant women regarding prenatal testing and determine whether patients receive sufficient information from healthcare providers to make informed decisions about undergoing or declining these tests. The analysis also included the frequency of various screening components (ultrasound examinations, combined tests, NIPT) and invasive procedures.

## 2. Materials and Methods

The study included 310 obstetric patients from the maternity wards of the Ludwik Rydygier Provincial Polyclinical Hospital in Toruń and the Kociewskie Health Center in Starogard Gdański, both located in northern Poland. Between 2021 and 2023, patients admitted to these wards were informed about the opportunity to participate in an anonymous survey and were provided with a questionnaire. The survey consisted of 37 open-ended and multiple-choice questions addressing obstetric history, demographic data, knowledge of indications, types, and procedures of prenatal tests, as well as sources of information on the topic. The reliability of the questionnaire was confirmed with a Cronbach’s alpha coefficient of 0.794. For selected questions, a key of correct answers was developed and subjected to statistical analysis. For example, the question “*which tests detect developmental defects in the fetus?*” included the following correct answers: ultrasound, prenatal tests, echocardiography, and genetic testing. For the question “*which tests detect genetic syndromes?*”, correct answers were: prenatal tests, combined test (FMF), ultrasound, non-invasive prenatal tests (NIPTs), and invasive procedures (e.g., amniocentesis). The recommended number of ultrasound examinations during pregnancy was considered to be at least four, in line with Polish and international guidelines, with particular emphasis on first-trimester diagnostics (11–14 weeks), second-trimester (18–22 weeks), and third-trimester scans (28–32 weeks).

Completed anonymous questionnaires were deposited by patients in a designated location, ensuring no external assessment. There were no strict exclusion criteria; however, only surveys completed by Polish-speaking adults were analyzed. Participation was voluntary, without restrictions on personal freedom or autonomy, and each respondent could complete the survey only once.

Data were compiled in Microsoft Excel (Windows 10). Statistical analysis was performed using Python (version 3.10.11) with the following libraries: pandas (2.2.1), matplotlib (3.8.3), seaborn (0.13.2), scipy (1.13.0), and pingouin (0.5.4). The significance level was set at *p* < 0.05.

## 3. Results

The study included 310 pregnant women, with a mean age of 31 years (range: 16–44). Among the 308 respondents who reported their age, 63 women (20.4%) were over 34 years old. Most participants had higher education (174 women, 56.9%), followed by secondary education (114 women, 37.3%) and primary education (17 women, 5.6%). A total of 273 women (88.1%) were professionally active, including 35 (12.8%) employed in healthcare-related fields. Regarding place of residence, 119 women (39.0%) lived in rural areas, 99 (32.5%) in small towns (up to 150,000 inhabitants), and 87 (28.5%) in large cities. For 124 women (40.3%), this was their first delivery; 122 (39.6%) had their second, and 62 (20.1%) had given birth three or more times. A history of one miscarriage was reported by 16.1% of respondents, while 8.1% had experienced two or more. Most women (278, 89.7%) delivered at term (≥37 weeks), whereas 32 (10.3%) had preterm deliveries. In terms of delivery mode, 184 women (59.4%) had vaginal births, and 126 (40.6%) underwent cesarean sections. The most common indications for cesarean section were fetal distress (28 patients, 22.3%), macrosomia and lack of labor progress (both 19 patients, 15.1%), and abnormal fetal position (15 women, 11.9%) (Table 1).

Among the newborns, 298 (97.1%) were genetically healthy and free of developmental defects. Nine infants (2.9%) presented health issues, including six (2.0%) with congenital anomalies. No genetic disorders were diagnosed. Reported anomalies included cardiovascular defects in four cases and urinary tract malformations in three, with one infant exhibiting both cardiac and urinary system defects. Half of these anomalies (three cases) were detected prenatally, while the remaining were diagnosed postnatally.

In the study group, 193 women (62.5%) experienced uncomplicated pregnancies. Among the 116 women (37.5%) with complications, the most frequently reported conditions were diabetes (39 women, 12.9%), thyroid disorders and infections (both 38 women, 12.6%), and hypertension (21 women, 7.0%).

Regarding knowledge assessment, 220 respondents (71.0%) correctly identified tests that detect developmental defects. The detailed distribution of responses is presented in Table 2.

A correct response to the question “*what tests detect genetic disorders in the fetus?*” was provided by 230 women (74.2%). The detailed distribution of responses is presented in Table 3.

A correct number of recommended ultrasound examinations during pregnancy was indicated by 158 women (51.0%). An incorrect number was provided by 152 women (49.0%), of whom 70 (46.1%) expressed confidence in their knowledge. Regarding the timing of subsequent ultrasound examinations, 94 women (30.3%) correctly identified the recommended timeframe for the first examination, 118 (38.2%) for the second, and 82 respondents (26.5%) for the third.

The actual number of ultrasounds performed during the most recent pregnancy was reported by 216 women. Among them, 138 patients (64.9%) stated that ultrasound was performed at every visit, although not all respondents specified the exact number of visits. On average, 5.8 ± 5.3 examinations were performed, with a median of 6 (range: 0–30). Three or more examinations were conducted for 178 patients (57.4%).

First-trimester ultrasound within the recommended timeframe was reported by 205 women (66.1%), second-trimester by 198 (63.9%), and third-trimester by 198 (63.9%).

Information regarding the timing of ultrasound examinations primarily came from the attending physician performing the scan (205 women, 66.1%) or from the referring physician (58 women, 19.7%). Overall, physicians were the main source of knowledge for 263 respondents (85.8%). Only 3 women (1.0%) reported receiving this information from a midwife or other medical staff. The remaining participants obtained information from family and friends, the Internet, or other sources (Table 4).

The range of conditions detectable by the first-trimester combined test was correctly identified by 198 women (63.9%). A correct set of examinations included in the combined test was reported by 127 women (41.0%). One hundred forty-five women (46.8%) accurately indicated the gestational period during which this test is performed; however, only 36 women (11.7%) correctly identified which women should undergo the test. As an indication for the test, maternal age over 34 years was reported by 112 women (36.1%), while another age threshold was indicated by 31 women (10.0%). A genetic history was noted by 102 women (32.9%).

Among respondents, 134 women (48.6%) underwent the first-trimester combined test. It was not specified whether the test was performed under the state-funded Prenatal Testing Program or privately. Of the women aged over 34 years, 37 (58.7%) underwent the test, compared to 97 respondents (38.5%) in the younger group. Among those who underwent the combined test, 64 women (56.6%) correctly identified the indication for the test.

As a source of knowledge influencing the decision to undergo the combined test, 130 respondents (97.0%) indicated a physician, while one patient (0.7%) cited a midwife or other medical staff, one (0.7%) mentioned family or friends, and another referred to scientific publications or other sources.

In the entire study population, 73 respondents (23.6%) indicated the physician who performed the test as a source of knowledge about the combined test, while 98 women (31.6%) cited the referring physician. Four patients indicated both responses. In total, 167 women (53.9%) identified a physician as their source of knowledge. Information obtained from a midwife or another staff member was cited by 10 patients (3.2%), from family or friends by 10 (3.2%), from the Internet by 17 (5.5%), and from other sources by 4 pregnant women (1.3%).

Regarding NIPT, 189 women (61.2%) stated that they did not know which conditions the test detects. Among those declaring knowledge, 106 women (87.6%) provided correct answers, and 86 (27.8%) correctly described the method of examination (blood draw). Ninety-four respondents (30.3%) incorrectly stated that ultrasound is included in NIPT, and 14 women (4.5%) indicated amniotic fluid testing.

One hundred forty-five women (46.8%) claimed to know when NIPT is best performed, but only 87 (28.1%) correctly specified the timing. Correct indications for NIPT were mentioned by 109 patients (35.2%). No respondent indicated high risk of chromosomal aberrations as an indication for NIPT; maternal age over 34 years was reported by 50 respondents (16.1%), another age threshold by 7 patients (2.3%), and a referral from the attending physician by 3 women (1.0%). Additionally, 5 respondents (1.6%) cited family history.

A total of 251 respondents answered whether they underwent NIPT during pregnancy. Of these, 29 women reported having the test. Among them, 13 (44.8%) were classified as having increased risk of chromosomal aberrations (one due to combined test results, others due to age ≥ 35 years). The percentage of patients under 35 who underwent NIPT was 8.7% (17 out of 195), while among those aged 35 and older, it was 22.2% (12 out of 54).

The referring physician was the source of knowledge about NIPT for 50 women (16.1%), while the physician performing the test was cited by 51 women (16.5%), totaling 99 women (31.9%) who identified a physician as their source. A midwife or other medical staff served as a source for 6 women (1.9%), family and friends for 18 women (5.8%), the Internet for 11 (3.5%), and other sources for 6 women (1.9%).

Knowledge regarding conditions detected after amniocentesis was reported by 135 respondents (43.5%). The most commonly indicated condition was trisomy 21, noted by 63 women (20.5%), followed by trisomy 18 (34 women, 11.0%) and trisomy 20 (13 women, 6.5%). The gestational age at which amniocentesis is recommended was correctly identified by 77 women (24.9%). A total of 195 women (62.9%) stated that they did not know the indications for amniocentesis. Correct indications were identified by 105 women (34.0%). Increased risk of chromosomal aberrations was noted as an indication by 62 respondents (20.0%), abnormal ultrasound findings by 39 women (12.6%), and advanced maternal age by 45 women (14.5%).

In the studied group, 5 women (1.8%) underwent amniocentesis or chorionic villus sampling during pregnancy. Due to the small size of this group, no further analysis was conducted.

In the next stage, respondents were categorized by knowledge level: high, medium, and low. The maximum score was 16 points. A low level of knowledge was assigned to 131 patients scoring below 4 points, medium to 139 women scoring 4–7 points, and high to 51 respondents scoring above 8 points. Statistical analysis revealed no significant correlation between age group (<34 years vs. ≥34 years) and knowledge level (linear correlation test, *p* = 0.434). Data are presented in Table 5.

Considering the education level of respondents, individuals with primary education demonstrated a significantly higher prevalence of low knowledge regarding prenatal testing compared to women with secondary or higher education. Conversely, the highest knowledge levels were observed among women with higher education. A statistically significant association between education level and knowledge about prenatal testing was confirmed (linear correlation test, *p* < 0.001). The data are presented in Table 6.

The relationship between knowledge level regarding prenatal tests and employment in healthcare was analyzed. Statistical testing did not confirm a significant association (linear correlation test, *p* = 0.073). The data are presented in Table 7.

The level of knowledge among women living in rural areas, small towns, medium-sized towns, and large cities was analyzed. Knowledge level increased with the population size of the locality, with the highest scores observed among women residing in large cities. A statistically significant association between place of residence and knowledge level was confirmed (linear correlation test, *p* = 0.001). The data are presented in Table 8.

The impact of the number of pregnancies (births and miscarriages) on knowledge level was analyzed. Women giving birth for the first time exhibited the highest level of knowledge, while knowledge decreased with the number of births. A statistically significant association between parity and knowledge level was confirmed (linear correlation test, *p* = 0.013). The data are presented in Table 9.

The impact of a history of miscarriage on knowledge level was analyzed. Statistical testing did not confirm a significant association (linear correlation test, *p* = 0.063). The data are presented in Table 10.

The impact of pregnancy complications on knowledge level was analyzed. Statistical testing did not confirm a significant association (linear correlation test, *p* = 0.100). The data are presented in Table 11.

It is particularly noteworthy that no correlation was found between the level of knowledge about prenatal tests and having previously given birth to a child with a congenital defect (linear correlation test, *p* = 0.665). The data are presented in Table 12.

Another aspect analyzed was the level of knowledge about prenatal tests among patients who underwent ultrasound examinations during pregnancy in accordance with recommended guidelines. This group included 198 women who had ultrasound examinations performed as advised. As anticipated by the authors, women who adhered to the recommended ultrasound schedule demonstrated a significantly higher level of knowledge (linear correlation test, *p* < 0.001).

In the multiple regression model, significant predictors of knowledge level included the number of previous pregnancies, place of residence, and level of education (Table 13).

When narrowing the multiple regression model to five significant factors—maternal age, number of previous pregnancies, mode of delivery, place of residence, and level of education—the results remained consistent. The strongest predictor of knowledge level was education, followed by place of residence and number of previous pregnancies. Maternal age and mode of delivery were not statistically significant. The data are presented in Table 14.

In the logistic regression analysis assessing awareness of prenatal testing (aware vs. unaware), the final model retained three predictors: maternal age, place of residence, and level of education. Level of education demonstrated the strongest association with awareness, followed by place of residence. Maternal age showed a weaker effect and was not statistically significant. The results are presented in Table 15.

**Figure 1:**
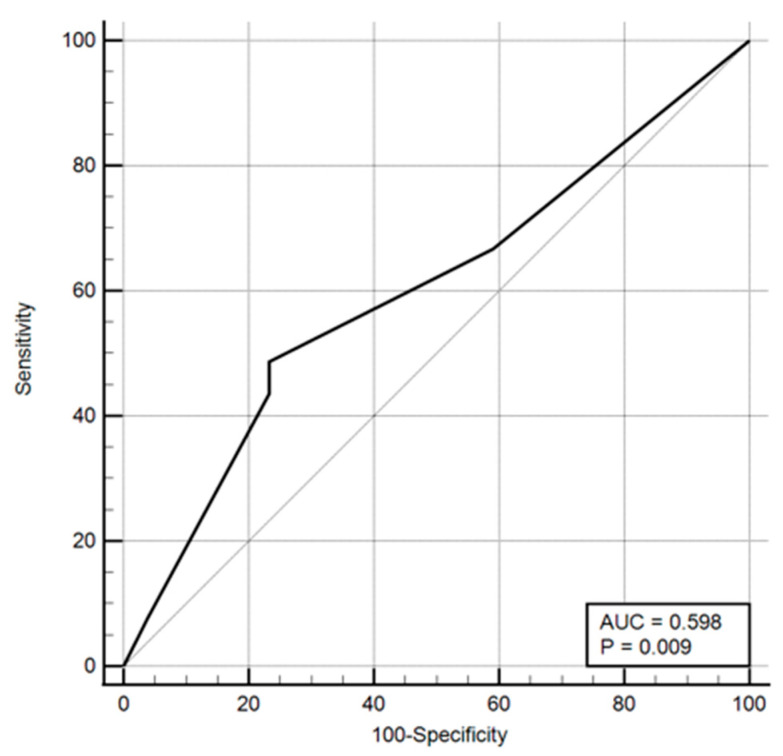
ROC curve including place of living.

**Figure 2:**
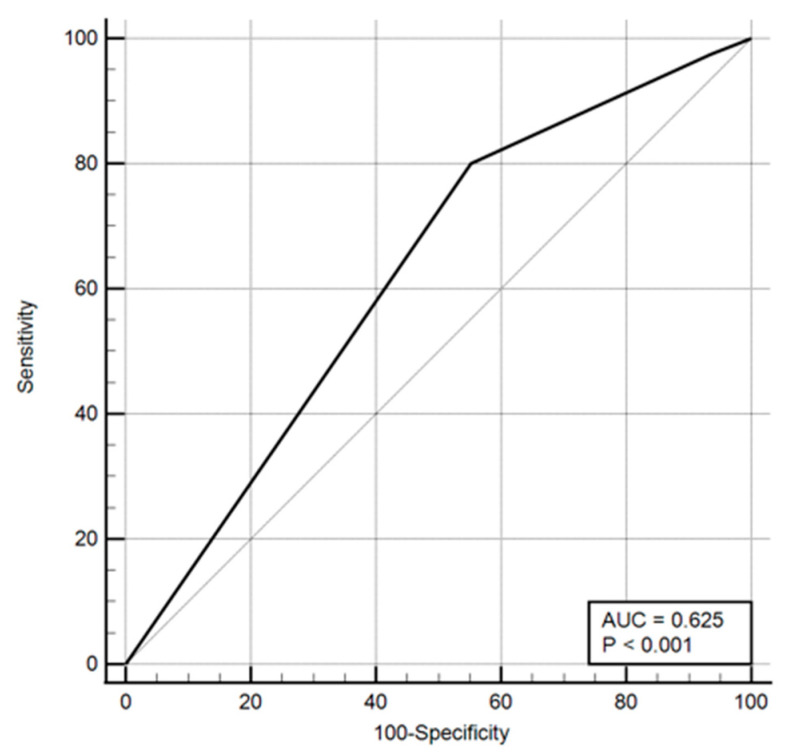
ROC curve including level of education.

**Figure 3:**
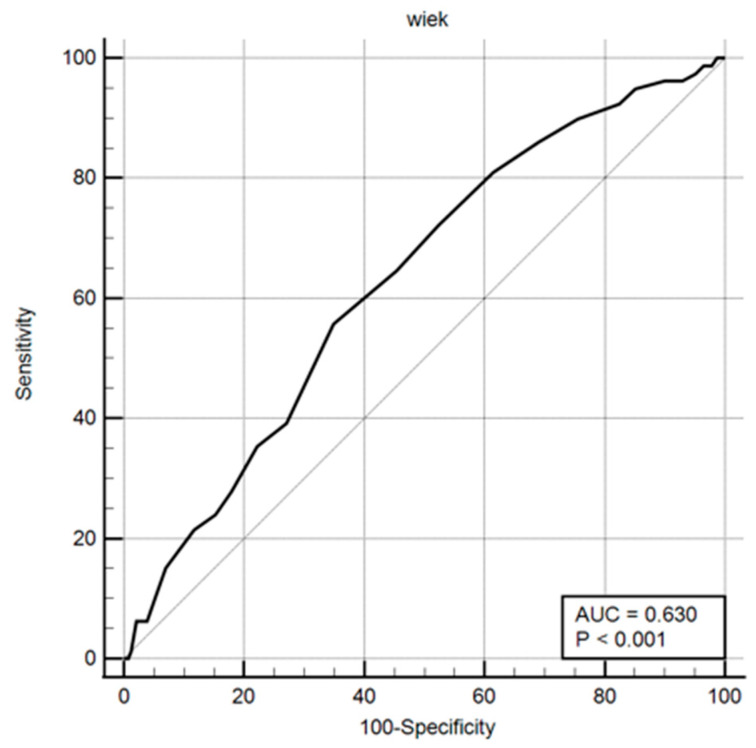
ROC curve including maternal age.

Odds ratios and ROC curves were computed for these parameters, with the results presented in both tabular and graphical formats (Table 16, Figure 1, Figure 2 and Figure 3).

Patients who correctly identified the recommended number of ultrasound examinations during pregnancy were significantly more likely to have undergone these examinations in accordance with clinical guidelines (Fisher’s exact test, *p* = 0.006). However, no significant difference was observed in knowledge regarding the appropriate gestational periods for first- and second-trimester ultrasounds between women who adhered to the standards and those who did not (Fisher’s exact test: *p* = 0.90 for first trimester; *p* = 0.553 for second trimester). In contrast, knowledge of the recommended timing for the third-trimester ultrasound reached statistical significance (Fisher’s exact test, *p* = 0.05), indicating that patients who correctly completed all ultrasound examinations were more likely to know when this examination should be performed.

The analysis also examined whether women who underwent the combined test demonstrated greater knowledge of prenatal screening compared to those who did not. A strong association was confirmed (linear correlation test, *p* < 0.001). Among the 134 respondents who completed the combined test, knowledge of its individual components was significantly higher: conditions detected (Fisher’s exact test, *p* < 0.001), test components (*p* < 0.001), and recommended timing (*p* < 0.001).

A similar pattern was observed for non-invasive prenatal testing (NIPT). Both overall knowledge (linear correlation test, *p* < 0.001) and specific knowledge—conditions detected (Fisher’s exact test, *p* < 0.001), test components (*p* < 0.001), timing and indications (*p* < 0.001)—were significantly higher among the 29 women who underwent NIPT compared to other respondents.

Conversely, the performance of amniocentesis was not associated with overall knowledge of prenatal testing (linear correlation test, *p* = 0.791), nor with knowledge of conditions diagnosed through invasive testing (Fisher’s exact test, *p* = 0.372) or appropriate timing and indications (*p* = 1.000). These findings should be interpreted with caution, as amniocentesis was performed in only five participants.

The study also assessed whether knowledge levels varied by the source of information. Sources were categorized into three groups: physicians (including primary care and specialists), other medical staff, and non-medical sources. Women who identified physicians as their primary source of information demonstrated significantly better alignment with guidelines regarding the number of ultrasound examinations (Fisher’s exact test, *p* < 0.001) and their timing: first trimester (*p* = 0.004), second trimester (*p* = 0.002), and third trimester (*p* = 0.001). No respondents reported other medical personnel as their source of ultrasound-related knowledge.

Similarly, patients informed by physicians correctly identified the conditions detected by the combined test (chi-square test, *p* < 0.001), its components (*p* < 0.001), and the appropriate gestational period (*p* < 0.001). Although the group informed by other medical staff was small (No = 7), these respondents also provided more accurate answers (*p* < 0.001).

In contrast, the source of information did not significantly influence knowledge regarding NIPT, including conditions detected (chi-square test, *p* = 0.885), test components (*p* = 0.749), timing (*p* = 0.712), or indications (*p* = 0.223).

## 4. Discussion

The range of prenatal tests offered to pregnant women depends on several factors. Qualification is typically based on information provided by the physician regarding the type of tests, their scope, and the recommended gestational period for their performance. After receiving this information, the patient decides which tests to undergo. This study aimed to assess whether pregnant women possess sufficient knowledge to make informed decisions about prenatal screening, identify the main sources of this knowledge, and determine which sources are perceived as most reliable. Additionally, the study examined awareness of tests recommended for women at high risk of congenital anomalies.

Survey results revealed that over 70% of respondents were aware that congenital anomalies—both structural and genetic—can be detected through ultrasound examinations. However, only about half correctly identified the recommended number of ultrasound scans and their timing during pregnancy. Analysis of actual practice suggested a tendency toward overuse of ultrasound, with many women reporting scans at nearly every visit, averaging six examinations instead of the recommended minimum of three [10]. Given that physicians were cited as the primary source of information by over 85% of respondents, these findings raise questions about whether misinformation stems from inadequate communication or from patients’ misunderstanding of indications for additional scans, such as fetal growth restriction or multiple pregnancies.

A similar knowledge gap was observed regarding the combined test. While 63% of respondents correctly identified its purpose, only 11.7% accurately indicated reimbursement criteria according to prevailing guidelines. The most frequently cited indication—maternal age over 34 years—was correctly identified by 36% of respondents. However, this criterion was not exclusive; the combined test was recommended for all pregnant women during the study period, with age serving only as a threshold for reimbursement. Physicians often cited lack of funding as a reason for not recommending the test [24].

The proportion of women aware of eligibility for the combined test was low, as reflected by the fact that only 48.6% of respondents underwent the test, despite guidelines requiring physicians to inform patients of this option. Among women over 34 years of age—who qualified for reimbursement—63.8% underwent the test. Whether non-participation resulted from insufficient information or personal choice remains unclear. Literature suggests that inadequate or unclear communication is a common reason for declining recommended examinations [25,26,27].

Non-invasive prenatal testing (NIPT) remains a relatively new, costly, and less commonly utilized diagnostic method, which contributes to limited patient awareness [28]. This was reflected in our findings, where respondents demonstrated significantly restricted knowledge about NIPT. Due to its high cost, lack of reimbursement, and limited dissemination of information, NIPT was rarely performed, even among women with clear indications [24]. Within our study population, women classified as high-risk for genetic disorders—specifically those aged 35 years or older—were nearly three times more likely to undergo NIPT compared to other respondents.

Both the combined test and NIPT were most frequently performed upon physician recommendation, underscoring the critical role of healthcare providers in shaping patient decisions. However, the low uptake of these tests suggests that current guidelines are not being communicated effectively. Alarmingly, women in high-risk groups did not exhibit greater knowledge about prenatal testing than other respondents. Whether this reflects insufficient counseling or unclear communication remains uncertain. Gates highlighted that patients respond more effectively to risk presented as frequency (e.g., 1:100) rather than percentage (1%) [26], suggesting that risk communication strategies may require refinement. Furthermore, it remains unclear whether women classified as intermediate-risk following the combined test are adequately informed about the availability of non-invasive diagnostic options [28].

Our study did not assess indications for invasive diagnostics or patient consent for amniocentesis. Previous research emphasizes the importance of how information about invasive procedures is conveyed and its influence on patient decision-making [29,30,31]. Sadłecki et al. similarly noted that accurate communication regarding the benefits and risks of both invasive and non-invasive tests is essential for informed consent [32].

Interestingly, no correlation was observed between knowledge level and professional involvement in healthcare. This suggests that prenatal testing is either underrepresented or treated superficially in medical education, despite its relevance to individuals who often plan pregnancies shortly after completing their training. Literature confirms that even healthcare professionals engaged in prenatal care frequently lack sufficient knowledge about these tests [33].

Our analysis did reveal associations between knowledge and factors such as education level, urban residence, and parity. Women with higher education, those living in large cities, and mothers with older children demonstrated greater awareness. Conversely, no correlation was found between knowledge and previous miscarriages, despite expectations that such patients would seek information on genetic and developmental causes of pregnancy loss. Lower educational attainment is consistently cited as a barrier to understanding medical information, highlighting the need for communication strategies tailored to patient comprehension [34,35,36].

Another noteworthy finding was the absence of correlation between knowledge and having a child with a congenital defect, likely due to the small number of such cases in our sample. Overall, adherence to recommended prenatal testing (ultrasound, combined test, NIPT) was significantly higher among women with greater knowledge, reinforcing the importance of patient education [37,38,39,40]. Given that physicians are the primary source of information, it is imperative that they clearly explain the purpose, timing, and implications of these tests. Our results indicate that physician-provided information about ultrasound and the combined test aligns more closely with current guidelines compared to other sources.

However, the lack of difference in NIPT-related knowledge between patients informed by physicians and those relying on other sources is concerning. This suggests gaps in physician awareness and underscores the need for targeted education. Professional societies have recognized this issue and continue to publish guidelines and resources aimed at improving both provider and patient education [41].

To address these gaps, we recommend developing an informational brochure summarizing current prenatal testing guidelines. This resource should be distributed during the first prenatal visit, enabling patients to review the information independently and prepare questions for subsequent consultations. Such materials offer objectivity and consistency, reducing variability introduced by individual physician preferences [42].

Despite advances in patient education and the emergence of digital resources, including podcasts, there remains a lack of accessible, reliable online content explaining prenatal diagnostic options in clear, patient-friendly language. This issue persists globally, not only in Polish-language resources [43].

Finally, survey-based research carries inherent limitations. In our study, respondents may have consulted external sources such as the Internet while completing the questionnaire. Additionally, women with lower education levels may have been less inclined to participate, potentially introducing selection bias.

## 5. Conclusions

This study demonstrates that pregnant women generally possess insufficient knowledge about screening prenatal tests, limiting their ability to make informed decisions regarding available diagnostic options. Physicians were identified as the primary source of information, emphasizing the critical role of gynecologists in patient education. As the most trusted and authoritative source, gynecologists should allocate more time to explaining indications, procedures, and implications of prenatal diagnostics during consultations.

To improve patient understanding, the development of a standardized informational guide summarizing current recommendations is strongly recommended. This resource should be provided at the first prenatal visit to ensure consistency and clarity.

Furthermore, the findings indicate gaps in physicians’ knowledge about non-invasive prenatal testing (NIPT), highlighting the need for targeted professional training in this area.

## Figures and Tables

**Table 1:** Indications for cesarean section based on the interview with the study group.

Indication for Cesarean Section	No.	%
Fetal distress	28	22.2
Fetal macrosomia	19	15.1
Lack of progress in labor	19	15.1
Abnormal position (breech, transverse)	15	11.9
Hypertension (including preeclampsia)	11	8.7
History of cesarean section or myomectomy	10	7.9
Abnormal fetal positioning	6	4.8
Tokophobia	3	2.4
Other	17	13.5

**Table 2:** Distribution of Correct Responses Regarding Tests for Detecting Developmental Defects (Multiple Answers Allowed).

Method of Detection	No.	%
Ultrasound	150	48.4
Prenatal testing	94	30.3
Fetal echocardiography	7	2.3
Genetic testing	3	0.1

**Table 3:** Distribution of Correct Responses Regarding Tests for Detecting Genetic Disorders in the Fetus (Multiple Answers Allowed).

Detection of Genetic Disorders	No.	%
Prenatal testing	136	43.9
Combined test (FMF)	94	30.3
Ultrasound	45	14.5
Non-invasive prenatal testing	21	6.8
Invasive tests (e.g., amniocentesis)	21	6.8

**Table 4:** Sources of Knowledge Regarding Ultrasound Examinations During Pregnancy.

Source of Knowledge	No.	%
Physician performing the examination	205	66.1
Physician referring for the examination	58	19.7
Family and friends	16	5.2
Internet	13	4.2
Midwife and other medical staff	3	1.0
Other sources	6	1.9

**Table 5:** Level of knowledge according to respondent age.

Level of Knowledge	≤34 Years	>34 Years
Low	105 (42.86%)	21 (32.31%)
Medium	106 (43.27%)	37 (56.92%)
High	34 (13.88%)	7 (10.77%)

**Table 6:** Level of knowledge according to respondent education.

Level of Knowledge	Primary Education	Secondary Education	Higher Education
Low	14 (82.35%)	60 (52.63%)	48 (27.59%)
Medium	2 (11.76%)	47 (41.23%)	93 (53.45%)
High	1 (5.88%)	7 (6.14%)	33 (18.97%)

**Table 7:** Level of knowledge according to workplace.

Level of Knowledge	Employment Outside Healthcare	Employment in Healthcare
Low	95 (39.92%)	7 (20.00%)
Medium	108 (45.38%)	22 (62.86%)
High	35 (14.71%)	6 (17.14%)

**Table 8:** Level of knowledge according to place of residence.

Level of Knowledge	Village	City up to 50,000	City 50,000–150,000	City 150,000–500,000	City Over 500,000
Low	53 (44.54%)	48 (50.53%)	0 (0.00%)	22 (30.56%)	2 (13.33%)
Medium	54 (45.38%)	38 (40.00%)	2 (50.00%)	33 (45.83%)	12 (80.00%)
High	12 (10.08%)	9 (9.47%)	2 (50.00%)	17 (23.61%)	1 (6.67%)

**Table 9:** Level of knowledge according to the number of births.

Level of Knowledge	1	2	3	≥4
Low	43 (34.68%)	49 (40.16%)	17 (40.48%)	15 (75.00%)
Medium	61 (49.19%)	59 (48.36%)	20 (47.62%)	3 (15.00%)
High	20 (16.13%)	14 (11.48%)	5 (11.90%)	2 (10.00%)

**Table 10:** Level of knowledge according to history of miscarriage.

Level of Knowledge	Previous Miscarriage	No Miscarriages in History
Low	105 (44.68%)	21 (28.00%)
Medium	99 (42.13%)	44 (58.67%)
High	31 (13.19%)	10 (13.33%)

**Table 11:** Level of knowledge according to the presence of pregnancy-related conditions.

Level of knowledge	No Conditions During Pregnancy	Presence of Conditions During Pregnancy
Low	87 (45.08%)	38 (32.76%)
Medium	81 (41.97%)	62 (53.45%)
High	25 (12.95%)	16 (13.79%)

**Table 12:** Level of knowledge according to the previous birth of a child with a congenital defect.

Level of Knowledge	No Defects in Previously Born Children	Previous Birth of a Child with a Congenital Defect
Low	120 (40.13%)	3 (50.00%)
Medium	139 (46.49%)	3 (50.00%)
High	40 (13.38%)	0 (0.00%)

**Table 13:** Multiple regression modeling.

Independent Variables	Coefficient	Std. Error	*p*
(Constant)	0.2592		
Delivery	−0.5786	0.2358	0.0147
Miscarriage	0.2996	0.2461	0.2246
Gestational age	−0.02661	0.08142	0.7440
Age	0.08981	0.04666	0.0553
Habitation	0.4500	0.1542	0.0038
Illnesses during pregnancy	0.4287	0.4869	0.3794
Healthy child	−0.01935	0.5698	0.9729
Vaginal delivery/cesarean section	−0.4599	0.4173	0.2714
Congenital genetic defects	1.2745	1.3675	0.3521
Developmental abnormalities	−0.3769	2.4042	0.8756
Developmental abnormalities—previous children	−0.6869	1.4964	0.6466
Previous cesarean section	1.0868	0.5523	0.0501
Education	1.2375	0.3607	0.0007
Occupation	0.8854	0.5980	0.1398

**Table 14:** Multiple regression modeling including maternal age, number of previous pregnancies, mode of delivery, place of residence, and level of education.

Independent Variables	Coefficient	Std. Error	*p*
(Constant)	−1.6034		
Age	0.1000	0.04336	0.0218
Delivery	−0.5546	0.2092	0.0085
Education	1.2825	0.3411	0.0002
Previous cesarean section	0.9264	0.5056	0.0679
Habitation	0.4767	0.1429	0.0010

**Table 15:** Logistic regression analysis.

Variable	Coefficient	Std. Error	*p*
Delivery	−0.16228	0.18332	0.3760
Miscarriage	−0.031980	0.18067	0.8595
Gestational age	−0.037811	0.061985	0.5419
Age	0.073939	0.035769	0.0387
Habitation	0.24755	0.11224	0.0274
Illnesses during pregnancy	0.47010	0.33879	0.1653
Healthy child	−0.19109	0.40598	0.6379
Vaginal delivery/cesarean section	−0.45428	0.31838	0.1536
Congenital genetic defects	0.80883	0.97366	0.4061
Developmental abnormalities	0.19695	1.51649	0.8967
Developmental abnormalities—previous children	−19.65763	9228.59370	0.9983
Previous cesarean section	0.27265	0.41063	0.5067
Education	0.70093	0.30532	0.0217
Occupation	0.22115	0.41881	0.5975
Constant	−3.50798	2.63144	0.1825

**Table 16:** Odds ratios including maternal age, place of living and level of education.

Variable	Odds Ratio	95% CI
Age	1.0669	1.0066 to 1.1308
Habitation	1.3371	1.0966 to 1.6304
Education	2.1195	1.2255 to 3.6658

## Data Availability

The data presented in this study are available on request from the corresponding author. The data are not publicly available due to privacy and ethical restrictions.
